# Regulatory T Cells and Their Prognostic Relevance in Hematologic Malignancies

**DOI:** 10.1155/2017/1832968

**Published:** 2017-12-21

**Authors:** Giovanni D'Arena, Candida Vitale, Marta Coscia, Agostino Festa, Nicola Matteo Dario Di Minno, Vincenzo De Feo, Michele Caraglia, Gioacchino Calapai, Luca Laurenti, Pellegrino Musto, Giovanni Di Minno, Daniela Fenoglio

**Affiliations:** ^1^Hematology and Stem Cell Transplantation Unit, IRCCS Cancer Referral Center of Basilicata, Rionero in Vulture, Italy; ^2^Division of Hematology, AOU Città della Salute e della Scienza di Torino, University of Torino, Torino, Italy; ^3^Department of Molecular Biotechnology and Health Sciences, University of Torino, Torino, Italy; ^4^Dipartimento di Biochimica, Biofisica e Patologia Generale, University of Campania “Luigi Vanvitelli”, Napoli, Italy; ^5^Department of Clinical Medicine and Surgery, Regional Reference Centre for Coagulation Disorders, “Federico II” University, Napoli, Italy; ^6^Department of Pharmacology, University of Salerno, Salerno, Italy; ^7^Department of Biomedical and Dental Sciences and Morphological and Functional Sciences, University of Messina, Messina, Italy; ^8^Hematology Institute, Catholic University of Sacred Heart, Rome, Italy; ^9^Scientific Direction, IRCCS Cancer Referral Center of Basilicata, Rionero in Vulture, Italy; ^10^Centre of Excellence for Biomedical Research, Department of Internal Medicine, IRCCS Azienda Ospedaliero Universitaria San Martino-IST-Istituto Nazionale per la Ricerca sul Cancro, University of Genoa, Genoa, Italy

## Abstract

Regulatory T cells (Tregs) have a fundamental function in monitoring the immune homeostasis in healthy individuals. In cancer and, in particular, in hematological malignancies, Tregs exert a major immunosuppressive activity, thus playing a critical role in tumor cell growth, proliferation, and survival. Here, we summarize published data on the prognostic significance of Tregs in hematological malignancies and show that they are highly conflicting. The heterogeneity of the experimental approaches that were used explains—at least in part—the discordant results reported by different groups that have investigated the role of Tregs in cancer. In fact, different tissues have been studied (i.e., peripheral blood, bone marrow, and lymph node), applying different methods (i.e., flow cytometry versus immunohistochemistry, whole blood versus isolated peripheral blood mononuclear cells versus depletion of CD25^+^ cells, various panels of monoclonal antibodies, techniques of fixation and permeabilization, and gating strategies). This is of relevance in order to stress the need to apply standardized approaches in the study of Tregs in hematological malignancies and in cancer in general.

## 1. Introduction

Regulatory T cells (Tregs) constitute a small-size subpopulation of CD4^+^ T cells, accounting for 1–4% of circulating CD4^+^ lymphocyte in humans, specialized in suppressive functions that control unwanted immune responses not only toward self-antigens but also toward foreign antigens in the context of the immune tolerance [1].

Gershon and Kondo from Yale University first proposed the existence of CD8^+^ T cells with suppressive activity more than 40 years ago [2]. However, after the initial great interest following this first report, due to the fact that a precise definition of Tregs lacked for several years, no further advances in the study of this cell population were made for decades. In 1995, Sakaguchi and coworkers identified Tregs in mouse as CD4^+^ T cells expressing surface interleukin-2 (IL-2) receptor *α*-chain (CD25) [3]. Baecher-Allan and coworkers, using flow cytometry and analyzing sorted cells *in vitro*, identified a very small subset of T cells with high expression of CD25 and regulatory function in humans [4]. However, CD25 is not exclusively restricted to Tregs, and its surface expression is also seen on effector T lymphocytes after activation [5]. The intracytoplasmic Forkhead helix box P3 (FoxP3), a transcription factor required for the development, maintenance, and function of Tregs was subsequently identified [6, 7]. The central role of this transcription factor is confirmed by the fact that a FoxP3 single gene mutation on the X chromosome induces in Scurfy mice a severe autoimmune/inflammatory disease. In humans, the same mutation causes a disease called IPEX (immune dysregulation, polyendocrinopathy, enteropathy, X-linked syndrome), characterized by autoimmune manifestations in multiple endocrine organs, such as diabetes and thyroiditis, inflammatory bowel disease, and severe allergies [8]. Finally, the absence of the heterodimeric IL-7 receptor (CD127) combined with CD4, CD25, and FoxP3, has been shown to better identify Tregs, avoiding the contamination from other cell populations such as activated effector T cells [9, 10].

## 2. Regulatory T Cells and Prognostic Significance in Cancer

The role of Tregs in cancer appears to be relevant by promoting tumor progression and suppressing effective antitumor activity [11–13]. Overall, the large majority of studies report that the frequency and the suppressive function of Tregs are increased in cancer patients as compared to healthy subjects. However, some issues are still a matter of debate, in particular the prognostic significance of this cell subpopulation. In general, Tregs predict poor outcome in cancer patients [12], but some reports have shown that higher Treg numbers and preserved activity are associated with a better prognosis [14–16].

This review stems from the need to reassess the topic of prognostic relevance of Tregs in cancer, focusing on patients with hematologic malignancies. For this purpose, we reviewed a large body of published papers conducting a PubMed literature search (keywords: Regulatory T cells, Hodgkin lymphoma, non-Hodgkin lymphoma, chronic lymphocytic leukemia, chronic myeloid leukemia, acute lymphoblastic leukemia, acute myeloid leukemia, multiple myeloma, monoclonal gammopathies, myelofibrosis, essential thrombocythemia, polycythemia vera, and Ph1-negative chronic myeloproliferative neoplasms).

## 3. Regulatory T Cells in Chronic Lymphocytic Leukemia

The accumulation of monoclonal B lymphocytes in the bone marrow, lymphoid organs, and peripheral blood is the hallmark of chronic lymphocytic leukemia (CLL), the most common form of leukemia in Western countries [17]. The importance of T cell dysregulation in the pathogenesis and development of CLL is now well established [18, 19], and in this setting, the role of Tregs has also been investigated [20, 21]. As shown in Table 1, several authors reported data on Tregs in CLL showing in the majority of cases an expansion of this population [22–31]. In addition, a correlation between higher Treg numbers and more aggressive clinical-biological features and adverse prognosis of CLL has been described.

As previously discussed [20], the reported percentage of Tregs in CLL is highly variable. According to the majority of reports, the percentage of Tregs is higher in CLL patients than in normal controls, and when the absolute number is considered, Tregs are always found to be significantly greater in CLL as compared to healthy donors.

Interestingly, based on their experimental work, Jak et al. speculated that the accumulation of Tregs in CLL is due to an increased proliferation induced by CD27/CD70 interaction in the lymph node proliferation centers and to a decreased sensitivity to apoptosis [22].

Dasgupta et al. tried to establish an optimal threshold level for prognostic purpose [28]. The cut-off was assessed by receiver operating characteristic (ROC) analysis. A cut-off of 5.7% and 35 cells/*μ*L for percentage and absolute Treg count, respectively, were determined as optimal in patients with CLL, along with a median Tregs percentage of 15.5% used to separate low- and high-risk patients. Using the same approach in the setting of Rai stage 0 CLL patients, our group found that the absolute number of Tregs was an independent predictor of time to the first treatment, with the best predictive cut-off being 41 cells/*μ*L [24]. Overall, these data show that the absolute Treg number is able to identify Rai stage 0 CLL patients at higher risk of requiring therapy.

Rissiek et al., applying a multidimensional scaling analysis to assess the composition of the circulating T cell populations, generated T cell scores showing that suppressive T cell profiles emerge early during monoclonal B cell lymphocytosis (MBL), the well-recognized pre-CLL stage [31–33]. As the disease evolves from MBL to overt and advanced CLL, specific sequential changes in T cells appear, progressively compromising the effector T cells function and contributing to disease progression [30].

In our hands too, the absolute number of Tregs in MBL patients was lower compared to CLL patients, but slightly higher than healthy controls [30]. In addition, the absolute Treg cell number directly correlated with more advanced CLL clinical stages and higher circulating B cell numbers. Of note, the absolute number of Tregs was lower in MBL patients as compared to early-stage CLL patients (0/A according to Rai/Binet stage). In summary, Treg numbers increase gradually from normal subjects to “clinical” MBL patients and are significantly higher in CLL patients as compared to MBL patients.

Regarding the functional properties, some authors reported a reduced inhibitory function of Tregs in CLL [27, 34]. On the contrary, Piper et al. showed that in CLL patients Tregs retain their function and are not influenced by chemotherapy [35]. A correlation between a higher circulating Treg numbers and dysfunctional V*γ*9V*δ*2 T cells in untreated CLL patients was also shown, thus corroborating the hypothesis that Tregs may not be only bystanders but have a functional role in this setting [36].

A normalization in Treg number was observed after fludarabine therapy [34], and also in CLL patients treated with lenalidomide, suggesting that such drugs are able to modulate cell-mediated immunity in CLL [37].

Finally, we also tested the ability of green tea, a popular beverage in China, Japan, and increasingly used in Western countries, to modulate Treg number in peripheral blood of CLL patients in the early phases of the disease, for which at the present time there is no effective intervention and a “wait and see” policy is generally adopted [38, 39]. We showed that the B cell lymphocyte count and the absolute circulating Treg number were reduced after 6-month consumption of oral green tea extract, suggesting that this compound can modulate circulating Tregs in CLL patients with early stage of disease and delay disease progression.

## 4. Regulatory T Cells in Lymphomas and Monoclonal Gammopathies

The neoplastic lymph nodes in Hodgkin lymphoma (HL) and non-Hodgkin lymphoma (NHL) contain not only neoplastic B cells but also nontumoral T cells, macrophages, and dendritic cells, constituting the so-called tumor microenvironment. The importance of the microenvironment in the pathogenesis and progression of lymphomas is still a matter of debate and many studies have focused on the role of its different components, including Tregs. Tregs are increased in lymphoma tissues and are able to inhibit cytotoxic CD8^+^ T cells exposed to lymphoma B cells [40].

Marshall et al. showed that HL-infiltrating lymphocytes are highly enriched in Tregs, which induce a profoundly immunosuppressive environment [41]. This was confirmed by Schreck et al. who demonstrated that in classical HL the microenvironment is dominated by Th2 cells and Tregs [42]. Moreover, a high ratio of Tregs over Th2 cells resulted in a significantly shortened disease-free survival.

However, conflicting results have been reported regarding the prognostic significance of Tregs infiltration in both HL and NHL. In fact, whereas in follicular lymphoma (FL), the most common form of low-grade NHL, germinal center (GC) diffuse large B cell lymphomas (DLBCL), and HL, an intrafollicular infiltration of Tregs, has a positive prognostic significance; this is not true in the case of non-GC-type DLBCL [43]. Moreover, as shown in Table 2, in some reports, a higher number of Tregs correlates with a good prognosis, while in other, it does not [43–49]. Of interest, Kim et al. evaluating Tregs on node biopsy of extranodal natural killer/T cell lymphomas showed that patients with poor performance status and with non-upper aerodigestive tract had a decreased number of Tregs (<50/0.40 mm^2^), while an increased number (>50/0.40 mm^2^) was associated with prolonged overall survival and progression-free survival [48]. Finally, Carreras et al. reported that the median Treg number in patients with FL at diagnosis had a median cell percentage of 10.5% [49]. Furthermore, patients were classified as having Tregs > 10%, 5–10%, and <5% with a 5-year overall survival of 80%, 74%, and 50%, respectively. Patients with transformed DLBCL showed lower Treg number with respect to patients with grades 1–3 FL.

Regarding the frequency and prognostic significance of Tregs, conflicting results have also been obtained in the field of monoclonal gammopathies (Table 3). In some reports, Tregs were found to be increased in frequency, while in others they were reduced or comparable with respect to healthy subjects [50]. Again, some authors reported a correlation with tumour burden and with worse prognosis, but this was not consistent among different publications [50–57]. We recently published our data on the flow cytometric evaluation of Tregs in multiple myeloma (MM) and monoclonal gammopathies of undetermined significance (MGUS) [51]. We found no differences in Treg frequency in MM and MGUS with respect to normal controls, and no correlations with main clinical and laboratory features in this disease setting were observed.

## 5. Regulatory T Cells in Acute Leukemias, Chronic Myeloid Leukemia, and Ph1-Negative Chronic Myeloproliferative Neoplasms

Few studies have been published regarding the role of Tregs in acute myeloid and lymphoid leukemias (Table 4) [58–61]. In a study by Bhattacharya et al., an increased number of Tregs was found in patients with B cell acute lymphoblastic leukemia (B-ALL), and a correlation with disease progression was highlighted [58].

Regarding chronic myeloid leukemia (CML), an interesting paper has been published by Zahran and Badrawy, in which Tregs were found increased in the peripheral blood of affected individuals as compared to controls. Moreover, Tregs frequency correlated with the level of BCR/ABL, basophil number, blast cell count, and Sokal score, and Treg number was higher in accelerated and blastic phase with respect to chronic phase [62]. Of note, Treg frequency declined after therapy with imatinib. Rojas et al. found a lower Treg number in patients who achieved a complete cytogenetic response [63], while higher Treg frequencies were found after stem cell transplant compared to normal controls and newly diagnosed patients [64]. Finally, the correlations with Sokal score and basophil number were validated by other studies [65, 66], whereas the impact of treatment has not been confirmed, since no changes in Treg frequency was observed after 6 months of tyrosine kinase inhibitors therapy [65].  Table 5 summarizes the results of studies analyzing Tregs in CML.

Hasselbalch et al. studied patients with Ph1-negative chronic myeloproliferative neoplasms and found that circulating Tregs were significantly expanded in patients treated with IFN-*α*2 with respect to healthy donors and in patients treated with hydroxyurea [66]. Kovacsovics-Bankowski et al. analyzed patients with polycythemia vera (PV) and essential thrombocythemia (ET) and found increased numbers of circulating Tregs and an enrichment in highly suppressive subsets (defined as CD39^+^/HLA-DR^+^) in patients treated with PegIFN-*α* with respect to those treated with hydroxyurea [67]. Moreover, molecular nonresponder patients showed a trend towards increased frequency of Tregs compared to responder patients, but no changes were observed in terms of absolute numbers of Tregs. Overall, a positive correlation between proliferating Tregs (Ki-67^+^), highly suppressive Tregs (CD39^+^/HLA-DR^+^), and JAK2^V617F^ allelic burden was found, thus suggesting that the lack of ability of PegIFN-*α* treatment to decrease circulating Tregs predicts a poor molecular response.

Primary myelofibrosis (PMF) is a clonal disease of the hematopoietic stem cell characterized by a variable degree of bone marrow fibrosis, splenomegaly, and an increased risk of leukemic transformation. Contradictory data about Tregs in PMF have been published (Table 6). Massa et al. reported a reduced frequency and absolute number of Tregs in PMF than in normal controls [68]. No association with clinical-biological features of the disease was found, but a correlation between reduced Treg frequency and longer disease duration in patients with CALR mutation genotype was described. In these patients, a higher Treg frequency is significantly associated with advanced disease, higher IPSS/DIPSS score, and lower hemoglobin concentration. The same authors later documented the effect of ruxolitinib on Treg frequency, showing that the treatment with this small-molecule JAK1/2 inhibitor leads to a profound and long-lasting reduction in the frequency of circulating Tregs [69]. Wang et al. found no significant differences in the number of Tregs in patients with primary or post-ET myelofibrosis [70]. However, they reported that ruxolitinib significantly inhibits the release of sIL2-R*α*, an inflammatory cytokine produced by Tregs, contributing to the clinical improvement of constitutional symptoms induced by the drug. These data have been further confirmed by an *in vitro* study in which the JAK1/2 inhibition by ruxolitinib was able to prevent Treg differentiation [71]. Table 6 summarizes the results of studies analyzing Tregs in Ph1-negative chronic myeloproliferative neoplasms.

## 6. Conclusions

Tregs have a fundamental function in maintaining the immune homeostasis in healthy individuals. In cancer and in particular in hematological malignancies, Tregs exert a major immunosuppressive activity, thus playing a critical role in tumor cell growth, proliferation, and survival. Published data on the prognostic significance of the Treg number in hematological malignancies show conflicting results. In our opinion, this variability reported by different groups is most likely explained by the heterogeneity of the experimental approaches that are used. In fact, different tissues have been studied (i.e., peripheral blood, bone marrow, and lymph node) and different analytic methodologies have been applied (i.e., flow cytometry versus immunohistochemistry). Moreover, while some authors studied the whole blood compartment, others evaluated the Treg population in isolated peripheral blood mononuclear cells or in a CD25-depleted subpopulation. Finally, various panels of markers, different techniques of fixation and permeabilization, and several gating strategies have been applied. This is of relevance to stress the need to apply standardized approaches in the study of Tregs in hematological malignancies and in cancer in general.

In perspective, in light of the increasing evidence of the important role of Tregs in immune evasion mechanism exerted by tumor cells, therapeutic interventions targeting intratumoral Treg infiltrates may be conceived in order to fight cancer. Treg inhibition or depletion, the latter uses monoclonal antibodies targeting surface antigens on Tregs such as CD25, is currently under investigation [72].

## Figures and Tables

**Table 1:** Most relevant published studies investigating frequency, function, and prognostic significance of Tregs in CLL.

Reference	Patients/controls evaluated	Samples tested	Marker panel used in Treg evaluation by flow cytometry	Treg frequency	Functional studies	Impact on prognosis
Beyer et al. [34]	CLL/controls	PB	CD4/CD25	Increased^∗^	Reduced inhibitory function	Extended disease (Binet stage)
Giannopoulos et al. [73]	CLL/controls	PB	CD4/CD25/FoxP3	Increased	Not performed	Binet stage
Jak et al. [22]	CLL/controls	PB	CD4/CD25/CD127	Increased	More resistant to drug-inducedapoptosis than controls	Not evaluated
D'Arena et al. [23, 24]	CLL/controls	PB	CD4/CD25/CD127	Increased with a gradual variation from normal subjects to clinical MBL to CLL	Not performed	Rai stage, lymphocytosis, LDH, first time to treatment
Weiss et al. [25]	CLL/controls	PB	CD4/CD25/FoxP3	Increased	Not performed	Unmutated IgVH, CD38, chromosomal aberrations
Lad et al. [26]	CLL/controls	PB and FNA	CD4/CD25/CD127/IL-10	Reduced both Treg and IL-10 expressing Treg; higher absolute number	Not performed	Correlation with LDT (Tregs but not CD45RA^+^ Tregs and CD8^+^ Tregs were lower in CD38^+^ZAP70^+^ CLL group (with respect to CD38^−^ZAP70^−^)
Biancotto et al. [27]	CLL/controls	PB	CD4/CD25/FoxP3	Increased	Slightly reduced suppressive activity	Correlation with ZAP-70 and CD38 expression
Dasgupta et al. [28]	CLL/controls	PB	CD4/CD25/CD127/FoxP3	Increased	Not performed	Correlated with ZAP70 and CD38 expression
Mpakou et al. [29]	CLL/controls	PB	CD4/CD25/CD127	Increased	Suppression of T effector cells	Advanced stage
D'Arena et al. [30]	Clinical MBL/CLL/controls	PB	CD4/CD25/CD127	Reduced as % but increased as absolute number with a gradual variation from normal subjects to clinical MBL to CLL	Not performed	
Rissiek et al. [31]	MBL/CLL/controls	PB	CD4/CD25/CD127/CD39	Expansion. Highly suppressive CD39^+^ Treg subset increased in all disease stages	Increasingly suppressive regulatory function initiating at MBL stage; effector function impairment after transition to CLL; partially recovered after chemo-immunotherapy	Shorter time to treatment

^∗^Increased at diagnosis; significantly reduced after fludarabine therapy. CLL: chronic lymphocytic leukemia; MBL: monoclonal B cell lymphocytosis; PB: peripheral blood; FNA: fine needle aspiration; LDT: lymphocyte doubling time.

**Table 2:** Most relevant published studies investigating the frequency and the prognostic significance of Tregs in lymphomas.

Reference	Patients/controls evaluated	Samples tested	Marker panel used in Treg evaluation by flow cytometry	Treg frequency	Functional studies	Impact on prognosis
Tzankov et al. [43]	Lymphomas	Node biopsy	FoxP3 (IHC)	Increased	Not performed	Correlation with disease-specific and failure-free survival in FL and disease-specific survival in germinal center-like DLBCL and OS and failure-free survival in classical HD, but negative prognostic effect in nongerminal center DLBCL. Independent prognostic significance for failure-free survival in classical HD and of borderline significance for OS in classical HD and disease-specific survival in germinal center-like and nongerminal center DLBCL
Alvaro et al. [44]	Classical HL	Node biopsy	FoxP3 (IHC)	Not reported	Not performed	Small number influenced negatively EFS and DFS
Schreck et al. [42]	Classical HL/hyperplastic tonsils	Node biopsy	FoxP3 (IHC)	Increased	Not performed	Increased DFS and EFS; high Tregs/Th2 ratio correlated with shortened DFS
Garcia et al. [45]	Gastric MALT lymphoma	Gastric biopsy	FoxP3 (IHC)	Increased with respect to DLBCL but similar to chronic gastritis	Not performed	Higher number correlated with response to antibacterial eradication therapy
Koreishi et al. [47]	Relapsed/refractory HL	Node biopsy	FoxP3 (tissue microarray)	Not reported	Not performed	Lower Tregs correlated with poor OS
Chang et al. [46]	DLBCL/normal	Node biopsy	CD4^+^ CD25^+^	Increased	Not performed	Higher with poor survival and IPI
Kim et al. [48]	Extranodal natural killer/T cell lymphoma	Node biopsy	FoxP3 (IHC)	Heterogenous expression	Not performed	The decreased number of Tregs was more common in patients with poor performance status or in those presented in nonupper aerodigestive tract. Patients with increased numbers of Tregs showed prolonged OS and PFS.
Carreras et al. [49]	FL at diagnosis and relapse	Node biopsy	FoxP3 (IHC)	Not reported	Not performed	Inversely correlated with OS. Patients with very low numbers of Tregs (<5%) presented more frequently with refractory disease. No correlation with FLIPI. Patients with transformed DLBCL had lower Treg percentages than FL grades 1, 2, or 3

HL: Hodgkin's lymphoma; FL: follicular lymphoma; DLBCL: diffuse large B cell lymphoma; MALT: mucosa-associated lymphoid tissue; IHC: immunohistochemistry; OS: overall survival; DFS: disease-free survival, EFS: event-free survival.

**Table 3:** Most relevant published studies investigating the frequency and the prognostic significance of Tregs in monoclonal gammopathies.

Reference	Patients/controls evaluated	Samples tested	Marker panel used in Treg evaluation by flow cytometry	Treg frequency	Functional studies	Impact on prognosis
Prabhala et al. [50]	MGUS/MM/controls	PBMC	CD4/FoxP3	Decreased	Unable to suppress anti-CD3-mediated T cell proliferation	Not evaluated
Beyer et al. [52]	MGUS/MM/controls	PBMC	CD4/CD25/FoxP3 (% of CD4^+^ cells)	Increased in MM versus MGUS (trend without statistical significance)	Strong inhibitory function	Not evaluated
Feyler et al. [53]	MGUS/MM/controls	PBMC and BM	CD4/CD25/FoxP3	Increased in PBMC but not in BM	Not evaluated	Correlation with disease burden (paraprotein)
Gupta et al. [54]	MM	PBMC	CD4/CD25/CD127/FoxP3 (% of CD4^+^ cells)	Reduced in untreated which increased after treatment with lenalidomide	Able to inhibit proliferation of CD4^+^CD25^−^ T cells	Increase of Tregs in responding patients to therapy; decrease correlation with ISS I + II
Muthu Raja et al. [55]	MGUS/SMM/MM	PB/BM whole	CD4/CD25/CD127/CD45RA (% of CD4^+^ cells)	Increased in MM but not in SMM and MGUS	Able to inhibit proliferation of CD4^+^ T cells and the secretion of IFN-γ	Correlation with adverse clinical features (hypercalcemia, lower normal PC, and IgA subtype); no correlation with ISS; predict time to progression; MM patients with ≥5% of Tregs had inferior time to progression
Giannopulos et al. [56]	MM/controls	PBMC	CD4/CD25/FoxP3	Increased	Not evaluated	Correlation with shorter overall survival
Foglietta et al. [57]	MM/MGUS/controls	Fresh PB and frozen BM	CD4/CD25/FoxP3	Similar	Effective suppressor function	No correlation with the pattern of BM infiltration
D'Arena et al. [51]	MM/MGUS/controls	PB whole	CD4/CD25/CD127 (% and absolute number)	Similar	Effective suppressor function	No correlation with laboratory and clinical variables; no correlation with outcome

MM: multiple myeloma; MGUS: monoclonal gammopathy of uncertain significance; SMM: smoldering multiple myeloma; ISS: international staging system; PB: peripheral blood; PBMC; peripheral blood mononuclear cells; BM: bone marrow.

**Table 4:** Most relevant published studies investigating the frequency and the prognostic significance of Tregs in acute leukemias.

Reference	Patients/controls evaluated	Samples tested	Marker panel used in Treg evaluation by flow cytometry	Treg frequency	Functional studies	Impact on prognosis
Bhattacharya et al. [58]	B-ALL	PBMC /BM	CD4/CD25/CD127/FoxP3	Decreased	Higher suppressive capability on CD4^+^CD25^−^ regulatory T cells than controls	Increased frequency with disease progression
Wu et al. [59]	B-ALL/T-ALL/controls	PB	CD4/CD25	Higher	Not performed	Not evaluated
Wang et al. [60]	AML/controls	PBMC /BM	CD4/CD25	Higher	Inhibition of proliferation and cytokine production (IL2, IFN-*γ*) of CD4^+^CD25^−^T cells; improved IL-10 production under coculture of both subsets with stimulation	Not evaluated
Idris et al. [61]	B-ALL/controls	PB and BM	CD4/CD25/CD127	Increased	Not performed	Correlation with age

ALL: acute lymphoblastic leukemia; AML: acute myeloid leukemia; PB: peripheral blood; BM: bone marrow; PBMC: peripheral blood mononuclear cells; IL: interleukin; IFN: interferon.

**Table 5:** Most relevant published studies investigating the frequency and the prognostic significance of Tregs in chronic myeloid leukemia.

Reference	Patients/controls evaluated	Samples tested	Marker panel used in Treg evaluation by flow cytometry	Treg frequency	Functional studies	Impact on prognosis
Zahran and Badrawy [62]	CML/controls	PB	CD4/CD25/FoxP3	Increased	Not performed	Correlations with the level of BCR/ABL, basophils and blast cells. Significantly higher in accelerated phase and blastic phase than in chronic phase and with high Sokal score. Reduction of Tregs after therapy with IM
Hus et al. [65]	CP CML/controls	PB	CD4/CD25/FoxP3	Increased	Not performed	Correlation with higher basophiles. No change in frequency after 6 months of TKI inhibitors
Bachy et al. [74]	CP CML/controls	CD4+ enriched PBMC cells	CD4/CD25/CD127/FoxP3	Increased in PB. Increased in BM of patients on IM compared to healthy volunteers.	No difference in inhibition	Correlation with Sokal risk score
Rojas et al. [63]	CP CML/controls	PBMC	CD4/CD25/CD127/CD62L/FoxP3	Lower in patients in complete cytogenetic response	Enhanced proliferative response to purified protein derivative	Not evaluated
Nadal et al. [64]	CP CML/controls	PBMC	CD4/CD25/CD127/FoxP3/CTLA-4	Higher frequencies after transplant than normal controls and newly diagnosed patients	Purified Tregs from SCT patients had a more potent suppressive activity than those from healthy volunteers	Not evaluated

CP: chronic phase; BM: bone marrow; IM: imatinib; PB: peripheral blood; PBMC; peripheral blood mononuclear cells; BM: bone marrow; SCT: stem cell transplant; IM: imatinib; TKI: tyrosine kinase inhibitor.

**Table 6:** Most relevant published studies investigating the frequency and the prognostic significance of Tregs in Ph1-negative chronic myeloproliferative neoplasms.

Reference	Patients/controls evaluated	Samples tested	Marker panel used in Treg evaluation by flow cytometry	Treg frequency	Functional studies	Impact on prognosis
Hasselbalch et al. [66]	PV ET PMF Controls	PBMC	CD4/CD25/CD127	Not increased	Inhibitory activity preserved	Marked expansion of Tregs in patients treated with IFN-*α*2 with than treated with hydroxyurea
Kovacsovics-Bankowski et al. [67]	PV ET	PB	CD4/CD25/FoxP3/Ki-67	Not reported	Not performed	Tregs (including highly suppressive CD39^+^ HLA-DR^+^) increase in patients treated with PegINF*α*
Massa et al. [68, 69]	PMF	PB	CD4/CD25/CD127/FoxP3	Reduced	Not performed	In patients with CALR mutation genotype association with longer disease duration and hemoglobin concentration

PV: polycythemia vera; ET: essential thrombocythemia; PMF: primary myelofibrosis; PB: peripheral blood; PBMC: peripheral blood mononuclear cells; CALR: calreticulin; IFN: interferon.
